# *Araneusbonali* sp. n., a novel lichen-patterned species found on oak trunks (Araneae, Araneidae)

**DOI:** 10.3897/zookeys.779.26944

**Published:** 2018-08-07

**Authors:** Eduardo Morano, Raul Bonal

**Affiliations:** 1 DITEG Research Group, University of Castilla-La Mancha, Toledo, Spain University of Castilla-La Mancha Toledo Spain; 2 Forest Research Group, INDEHESA, University of Extremadura, Plasencia, Spain University of Extremadura Plasencia Spain; 3 CREAF, Cerdanyola del Vallès, 08193 Catalonia, Spain Ecological and Forestry Applications Research Centre Catalonia Spain

**Keywords:** DNA barcoding, Iberian Peninsula, isolated trees, mimicry, molecular phylogeny, *
Quercus ilex
*

## Abstract

The new species *Araneusbonali* Morano, **sp. n.** (Araneae, Araneidae) collected in central and western Spain is described and illustrated. Its novel status is confirmed after a thorough revision of the literature and museum material from the Mediterranean Basin. The taxonomy of *Araneus* is complicated, but both morphological and molecular data supported the genus membership of *Araneusbonali* Morano, **sp. n.** Additionally, the species uniqueness was confirmed by sequencing the barcode gene cytochrome oxidase I from the new species and comparing it with the barcodes available for species of *Araneus*. A molecular phylogeny, based on nuclear and mitochondrial genes, retrieved a clade with a moderate support that grouped *Araneusdiadematus* Clerck, 1757 with another eleven species, but neither included *Araneusbonali* sp. n. nor *Araneusangulatus* Clerck, 1757, although definitive conclusions about the relationships among *Araneus* species need more markers examined and a broader taxonomic coverage. The new species was collected on isolated holm oaks and forest patches within agricultural landscapes. Adults were mostly trapped on tree trunks, where their lichen-like colours favour mimicry, while juveniles were collected on tree branches. Specimens were never found either in ground traps or grass samples. This species overwinters as egg, juveniles appear in early spring, but reproduction does not take place until late summer-early autumn. *Araneusbonali* Morano, **sp. n.** was found in the same locality from where another new spider species was described. Nature management policies should thus preserve isolated trees as key refuges for forest arthropods in agricultural landscapes, as they may be hosting more unnoticed new species. After including *Araneusbonali* Morano, **sp. n.** and removing doubtful records and synonymies, the list of *Araneus* species in the Iberian Peninsula numbers eight.

## Introduction

The genus *Araneus* Clerck, 1757 includes 641 species of orb weaver spiders distributed worldwide (World Spider Catalog 2018), of which ten species have been cited in the Iberian Peninsula (Morano et al. 2014; Nentwig et al. 2018). It initially comprised species now included in different genera such as *Araniella* Chamberlin & Ivie, 1942, *Larinioides* Chamberlin & Ivie, 1942 or *Aculepeira* Caporiacco, 1934. While copulatory organs of the species in the younger genus are quite uniform, those of the species remaining in *Araneus* are highly heterogeneous (Dondale et al. 2003), suggesting that the genus is still far to form a natural grouping. The genus *Araneus* has never been revised and currently constitutes a melting pot of species that superficially resemble the type species *Araneusangulatus* Clerck, 1757. For example, some species described from Australia most likely do not really belong to *Araneus* (see Framenau 2012). Moreover, recent studies based on analyses of species from North America, Europe, and Australia have shown that *Araneus* is polyphyletic (Gregoric et al. 2015), which has been further corroborated by recent phylogenomic studies (Kallal et al. 2018).

Here, a new species, *Araneusbonali* sp. n. (Araneae, Araneidae), collected at several localities in western and central Spain is described. The comparison of *Araneusbonali* with reference material from the Iberian Peninsula and the south-western Mediterranean Basin available at the National Museum of Natural Sciences (Madrid) confirmed that the specimens represent a new species. In addition, a bibliographic review of the descriptions of Holarctic *Araneus* species was performed (Levi 1971, 1973, 1991; Dondale et al. 2003, Simon 1929; Nentwig et al. 2017; Grasshoff 1968; Šestáková et al. 2009, Yin et al. 1997, Tanikawa 2007, 2009). A special focus was paid to the species catalogues from northern Africa, where the spider fauna is still insufficiently known. In total seven species of *Araneus*, three of which are doubtful, have been cited in five countries: Egypt (Audouin 1826; Cambrigde 1876; Denis 1945), Tunisia (Pavesi 1880; Simon 1885), Lybia (Cambridge 1872; Simon 1908; Caporiacco 1934), Algeria (Lucas 1846; Simon 1899) and Morocco (Thorell 1875; Simon 1909; Denis 1945; Jocqué 1997).

Due to the challenging taxonomy of *Araneus*, the generic delimitation of the new species was queried by using morphological and molecular data. For the morphological analyses, the criteria exposed in the cladistic analysis of the family Araneidae performed by Scharff and Coddington (1997) were followed. For the molecular analyses a nuclear gene (28SrRNA) was sequenced and blasted in GenBank to assess the closer genera in terms of raw sequence similarity. Additionally, a fragment of the barcoding mitochondrial gene cytochrome oxidase I was sequenced and compared with the sequences of *Araneus* available in GenBank and BOLD (Barcoding of Life Datasystem). All the evidence pointed to the same conclusion: a new species of the genus *Araneus* had been collected.

Most of the specimens of the new species were collected during an extensive sampling campaign carried out in central Spain, in the same site where a new Eutichuridae spider was recently described, namely *Cheiracanthiumilicis* (Morano & Bonal, 2016). As in the case of *C.ilicis*, *Araneusbonali* sp. n. was found on the branches and trunks of holm oaks *Quercusilex* interspersed within agricultural fields. Based on a one-year-long systematic sampling, the habitat selection and the phenology of the new species were analysed, testing whether juveniles and adults showed different patterns. Finally, the literature was consulted and all the *Araneus* species cited from the Iberian Peninsula listed. Their taxonomic status is discussed and the number of species updated after removing dubious species records and including *Araneusbonali* sp. n.

## Materials and methods

### Study area

**Map 1.**
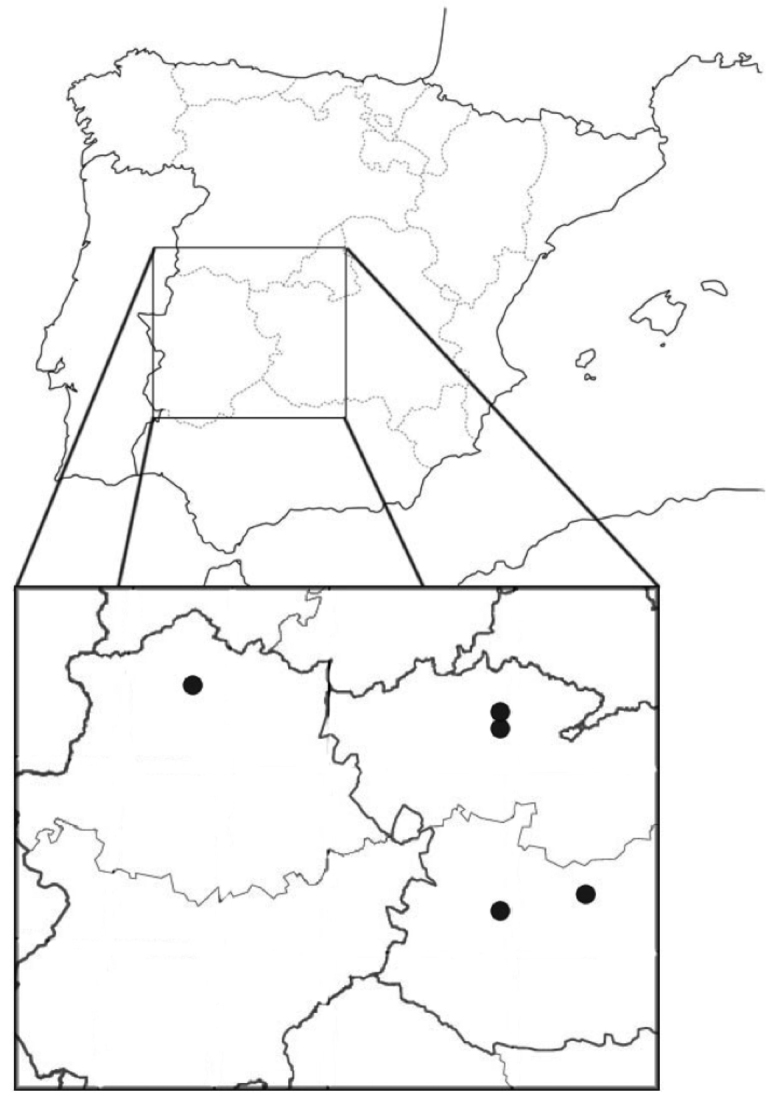
Geographic location of the sampling localities in the Iberian Peninsula.

Intensive spider sampling (see Morano and Bonal 2016 for details) was carried out in the locality of Huecas, province of Toledo, central Spain (coordinates lat. 39.994°N, long. -4.216°W; elevation 581 m a.s.l.), from September 2012 to September 2013 and collecting once a month. The climate in Huecas is dry Mediterranean, with a marked summer drought in which temperatures may reach 40°C and a precipitation of 365 mm per year. The study area extends over 900 Ha of flat agricultural landscape, with isolated oak trees and oak forest plots interspersed within a matrix of grasslands and cereal fields (see Bonal et al. 2012 for a detailed description). Tree density in the forest patches ranges from 20 to 50 trees per hectare, whereas the distance between isolated holm oaks ranges from 40 metres to more than 2 kilometres. Besides this systematic sampling, small numbers of spiders in other localities of central and western Spain were occasionally collected: Piedrabuena, province of Ciudad Real (lat. 39.041°N long. -4.230°W), Parque Nacional de Las Tablas de Daimiel, province of Ciudad Real (lat. 39.167°N long. -3.661°W) and Dehesa Casablanca, Guijo de Granadilla, province of Cáceres (lat. 40.077°N long. -6.097°W) (see Map 1).

### Sampling procedure

In Huecas, four different microhabitats were sampled: tree branches, tree trunks, grasses, and soil, at 23 holm oaks randomly selected (both isolated and within forest patches). The canopy of each tree was split into four parts based on the four cardinal points, a beating tray was placed under the canopy, and the branches of the corresponding canopy quarter beaten six times with a wooden stick. All the spiders falling on the beating tray were collected. Trunk traps consisted of a mosquito net attached to the tree trunk that trapped the spiders climbing up and eventually marching into the net. The net partially covered the trunks and ended in an inverted cone with a dry bottle on the top in which the spiders were collected. Soil spiders were caught in pitfall traps placed in pairs, one pair below the oak canopy and another pair at open grassland 10–15 metres far from the tree. These traps consisted of a cone through which ground-living spiders fell into a bottle filled with 90% ethanol and 10% glycerine to preserve the specimens. A small plastic roof was placed on each pitfall trap to protect them from direct sunlight and prevent alcohol evaporation. Lastly, in the grassland close to each study tree, the grass was swept for spiders with a sweep net along two 10 metres long transects on both sides of the straight line joining the two pitfall traps. Each specimen was placed in an Eppendorff tube tagged with the collection data and filled with alcohol 96% to preserve them for further morphological and molecular analyses.

### Morphological analyses

The epigynes of the females were extracted, cleaned, and mounted on slides for further analysis of the internal genitalia. In males one palp was extracted and illustrated; the palpal organ was expanded after maceration in lactic acid. Genitalia were preserved in microvials together with the specimen. The spiders were inspected with a Meiji EMZ-5 stereomicroscope, equipped with a Canon EOS 350D camera to take photos that were further used as templates to draw pictures of the specimens and their copulatory organs. The holotype and paratypes were deposited in the collection of the National Museum of Natural Sciences (**CSIC**), Madrid, Spain (**MNCN** collection of non-insect arthropods).

### Abbreviations

Eyes:

**ME** median eyes;

**LE** lateral eyes;

**ALE** Anterior lateral eye(s).

**AME** Anterior median eye(s).

**PLE** Posterior lateral eye(s).

**PME** Posterior median eye(s).

Female epygine:

**bs** basal epigynal plate;

**co** copulatory openings;

**ct** copulatory ducts;

**sc** scape;

**spt** spermathecae.

Male palp:

**c** conductor;

**cy** cymbium;

**dh** distal hematodocha;

**e** embolus;

**fe** femur;

**ma** median apophysis;

**p** patella;

**r** radix;

**s** stipes,

**sta** subterminal apophysis;

**t** tegulum;

**ta** terminal apophysis,

**ti** tibia.

All the measurements are given in millimetres.

### Molecular analyses

To corroborate that *Araneusbonali* sp. n. is not any of the recorded species, the DNA of three specimens (one male, one female and a juvenile) was extracted following the Aljanabi and Martínez (1997) salt extraction protocol. Due to the challenging taxonomy of *Araneus* species, the genus membership of the new species had to be confirmed in the first place. To do so a fragment (311 bp) of the nuclear ribosomal gene 28SrRNA was amplified using the primer pair (28Sa: GACCTGCCTTGAAACACGGA; 28Sb: TCGGAAGGAACCAGCTTACTA) (Whiting et al. 1997). This gene was chosen because of its low mutation rate, which makes it more appropriate for assessing deep phylogenetic relationships, for example among genera. Sequence chromatograms were assembled and edited using Sequencher 4.6 (Gene Codes Corp., Ann Arbor, MI, USA). The 28S sequences of the new species were blasted in GenBank to identify which were the closest species in terms of raw sequence similarity (i. e. proportion of identical nucleotides divided by the total number of nucleotides compared). In addition, a 28S gene tree was built to assess the position of *Araneusbonali* sp. n. with respect to the species of *Araneus* and other araneid genera (41 species) within the first 100 records retrieved after blasting the 28S of the new species in GenBank. All the sequences were aligned using MUSCLE (Edgard 2004) and the gene tree inferred using Bayesian inference analyses defining GTR + Invariants + Gamma evolutionary model, as implemented in Mr Bayes 3.2 software (Ronquist et al. 2012). The non-araneid orb-weaver *Tetragnathaextensa* (Tetragnathidae) was used as out-group.

Additionally, the barcoding fragment of the mitochondrial gene cytochrome oxidase I (cox1) was amplified using the primer pair HCO/LCO (Folmer et al. 1994). Sequence chromatograms were assembled and edited using Sequencher 4.6 (Gene Codes Corp., Ann Arbor, MI, USA). The cox1 sequences available for species of *Araneus* were downloaded from either GenBank or BOLD (Barcoding of Life Datasystem) (a total of 41 species; Accession Codes in Table 3), then combined with those of *Araneusbonali* sp. n. finally all of them aligned using MUSCLE (Edgard 2004) as implemented in MEGA7 software (Kumar et al. 2016). The uncorrected intra-specific genetic divergence was calculated within the three specimens of *Araneusbonali* sp. n. and the uncorrected inter-specific divergence between *Araneusbonali* sp. n. and the available *Araneus* species. (Table 3).

Phylogenetic relationships of the new species and the Holartic *Araneus* species were inferred by concatenating the available nuclear 28SrRNA and mitochondrial cox1 sequences (Accession Codes in Table 3). The focus was placed on the Holartic species because previous studies have shown that *Araneus* species from the southern hemisphere (e.g., Australia), most likely do not belong to the genus (Framenau 2012). The aligned concatenated matrix had a length of 944 base pairs (633 bp mitochondrial cox1, 311bp nuclear 28SrRNA). The best tree was inferred in a Bayesian framework as implemented in Mr Bayes 3.2 software (Ronquist et al. 2012). The species *Argiopeaemula* (Walckenaer, 1841) (Araneidae) was used as out-group. The best nucleotide substitution model for each gene was determined with jModelTest 0.1.1 (Posada 2008). Two parallel runs of ten million generations each were conducted using one cold and two incrementally heated Markov chains (Λ = 0.2), sampling every 1,000 steps. The standard convergence diagnostics implemented in MrBayes and the average standard deviation of the split frequencies were checked to assess whether the Markov chain had reached stationary. After 500,000 generations, the average standard deviation of the split frequencies stabilized in values close to zero (0.001). The trees sampled were summarized using the all compatible consensus command with 25% burn-in.

### Statistical analyses

Habitat preferences (tree branches, trunks, grass, and soil) of *Araneusbonali* sp. n. were analysed by comparing the proportion of individuals captured at each habitat. Because of the different sampling methods employed, the number of *Araneusbonali* sp. n. individuals captured at each habitat at random is not expected to be the same. Therefore, the proportion of the whole sample of spiders (all species) collected at each habitat was used as expected frequencies.

To assess habitat distribution and phenological differences between adults and juveniles Chi-square tests were used. In the case of phenology, the year was divided in four trimesters starting from January 1^st^ to assess whether the proportion of juveniles and adults differed among these periods. The correlation between the number of individuals and canopy surface (m^2^) was tested using a GLM (Generalized Linear Model, Poisson distribution, Logistic link function). Finally, a Mantel test was used to assess whether the spatial distance (in metres) between trees was correlated with the differences in the number of individuals collected.

## Taxonomy

### Araneus bonali

Taxon classificationAnimaliaAraneaeAraneidae

Morano sp. n.

http://zoobank.org/EC47CE37-07D2-4957-A28E-F989CCBFBCF6

**Figure 1.**
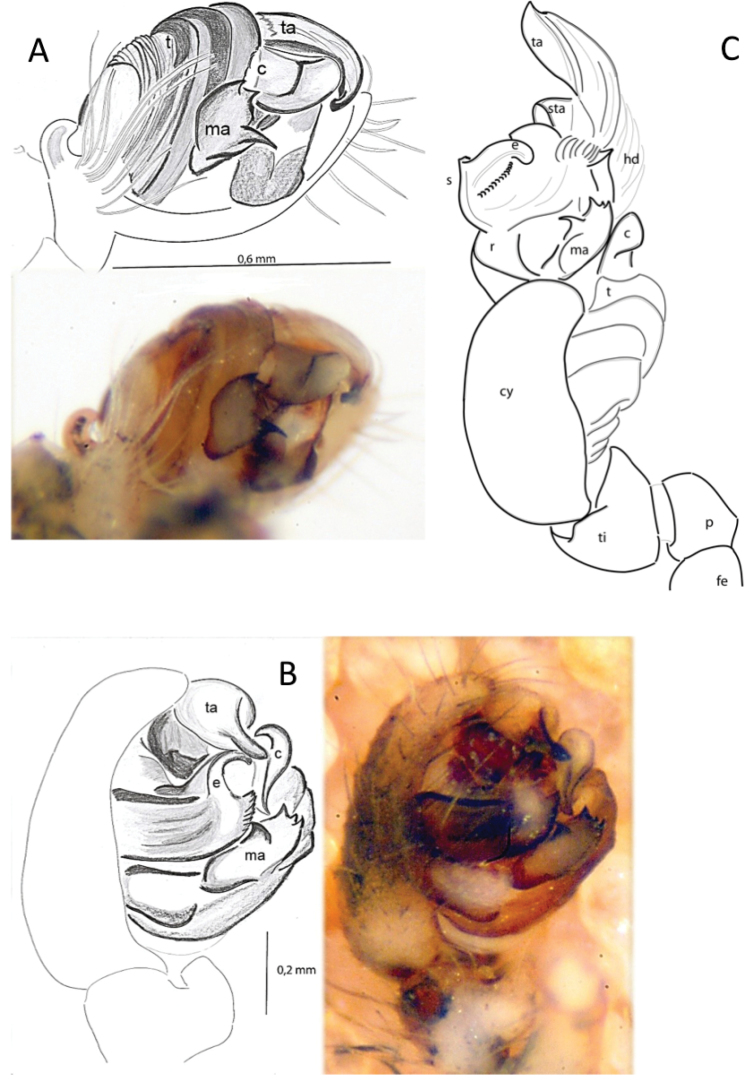
Right palp of *Araneusbonali* sp. n. **A** ventral **B** mesal **C** expanded pedipalp. Abbreviations: c – conductor; cy – cymbium; dh – distal hematodocha; e – embolus; fe – femur; ma – median apophysis; p – patella; r- radix; s – stipes, sta – subterminal apophysis; t – tegulum; ta – terminal apophysis, ti – tibia.

**Figure 2.**
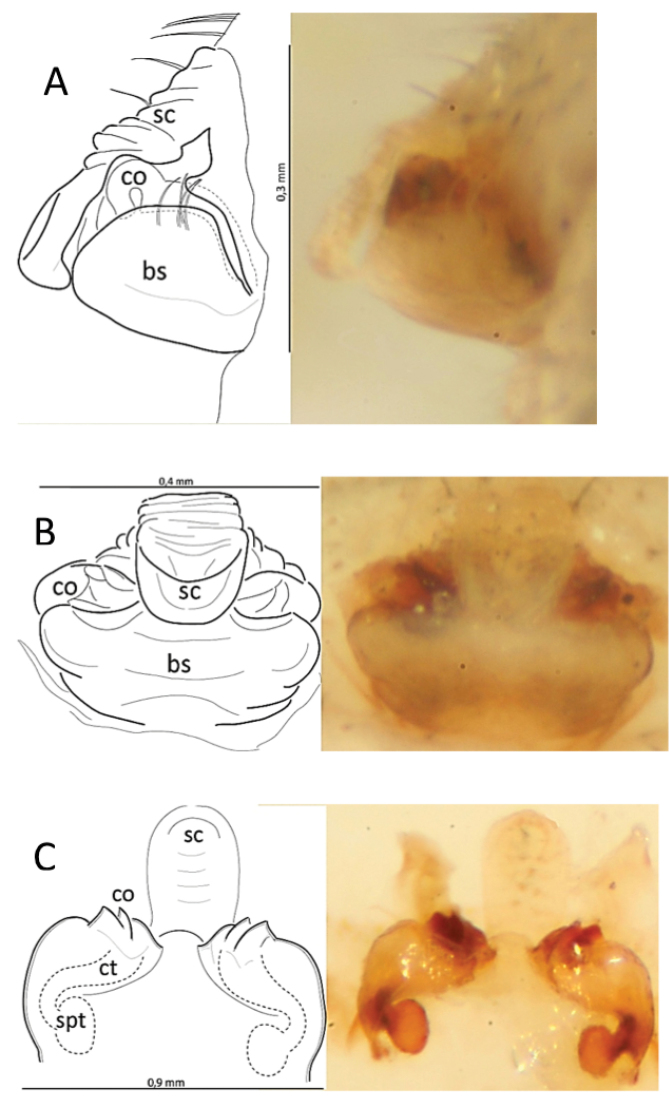
Epigyne and vulva of *Araneusbonali* sp. n. **A** epygine lateral **B** idem, ventral **C** vulva, posterior. Abbreviations: bs – basal plate; co – copulatory opening; ct – copulatory tube; sc – scape; spt – spermatheca.

**Figure 3.**
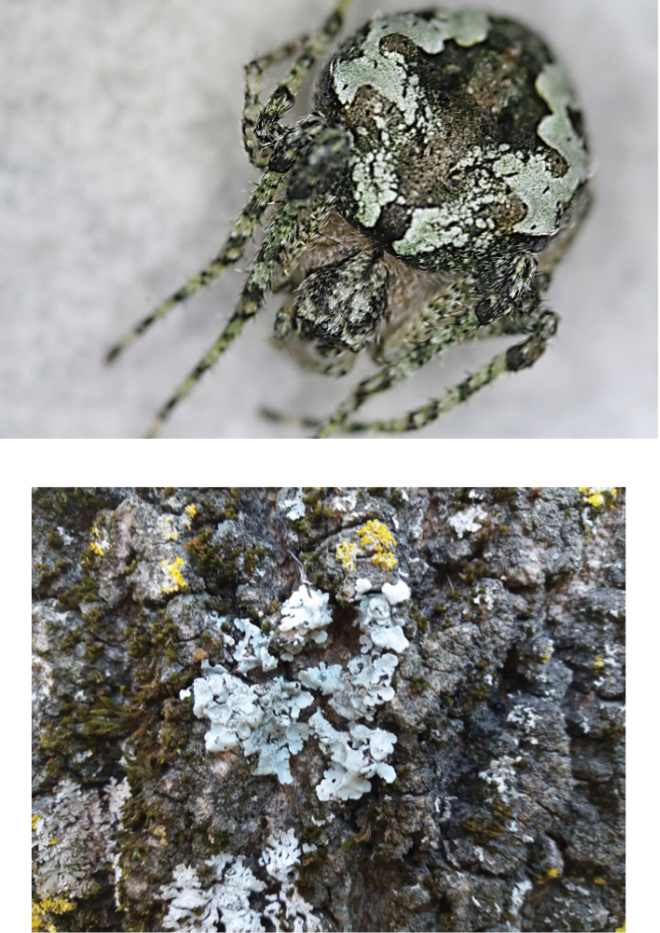
Images of an *Araneusbonali* sp. n. female and the surface of an oak trunk showing the lichen mimicry of this spider species.

**Figure 4.**
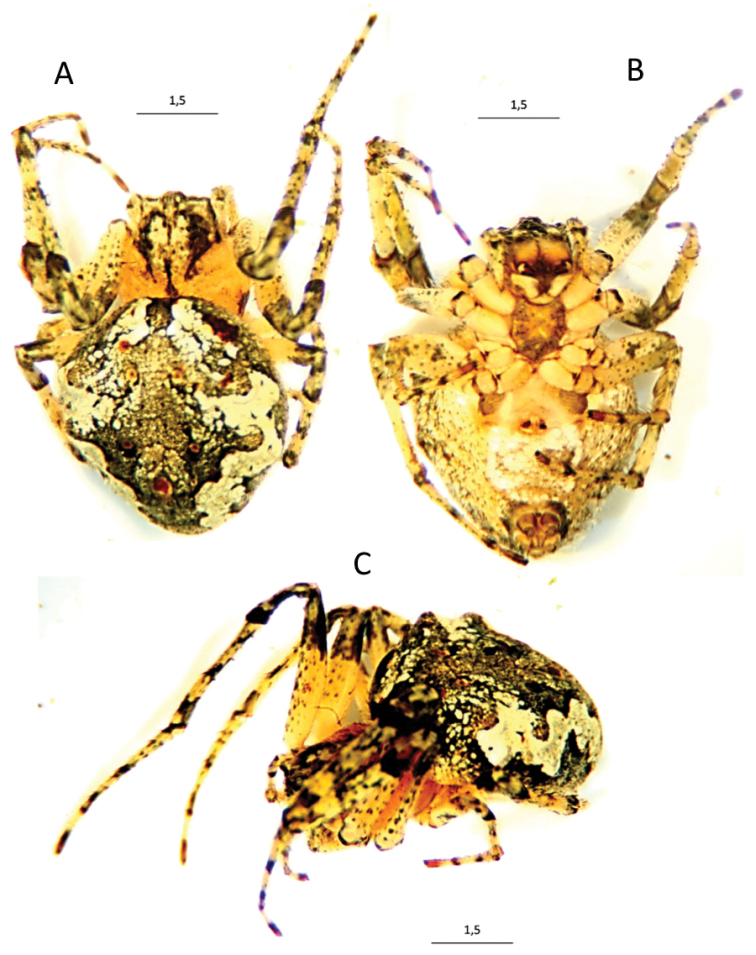
*Araneusbonali* sp. n., female under stereoscopic binocular microscope. **A** dorsal view **B** ventral view **C** lateral view (dimensions in mm).

**Figure 5.**
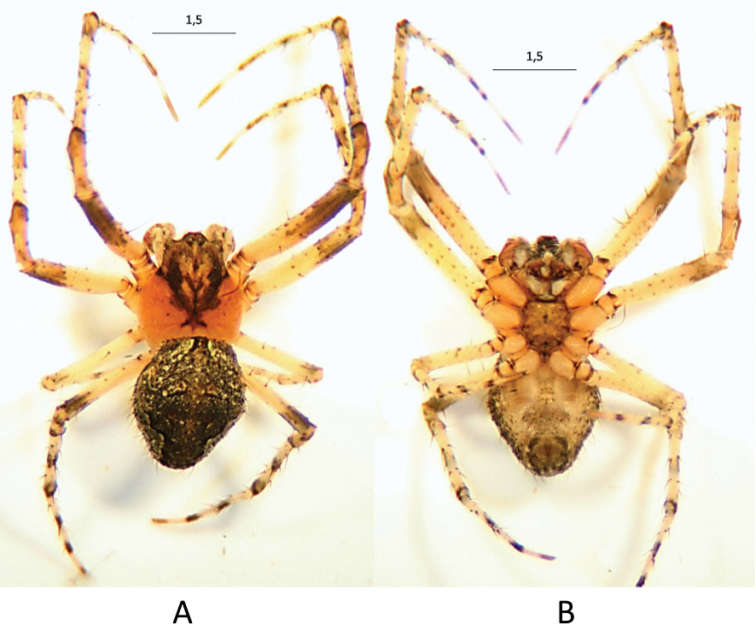
*Araneusbonali* sp. n. male under stereoscopic binocular microscope. **A** dorsal view **B** ventral view (dimensions in mm).

Figures 1 , 2 , 3 , 4 , 5

#### Material.

***Holotype.*** Female holotype collected by E. Morano in Spain (Map 1): Huecas (Toledo), 581 metres above sea level (lat. 39.994°long. -4.216°). 27 Sep 2013 (collected by branch beating) (voucher number MNCN 20.02/17497, National Museum of Natural Sciences (CSIC), Madrid, Spain).

***Paratypes.*** Collected in the same locality than the holotype but on different dates, the following 4 males & 3 females. Coordinates and voucher numbers are shown: 1♂, 25 Sep 2012 (trunk traps), 570 m.a.s.l. (lat. 40.013°long. -4.213°) (MNCN 20.02/17499); 1♀, 15 Oct 2012 (beating), 548 m.a.s.l. (lat. 39.994°long. -4.216°) (MNCN 20.02/17504); 1♂, 22 Oct 2012 (trunk traps), 548 m.a.s.l. (lat. 39.994°long. -4.216°) (MNCN 20.02/17500); 1♂, 30 Oct 2012 (trunk traps), 548 m.a.s.l. (lat. 39.994°long. -4.216°) (MNCN 20.02/17501); 1♀, 31 Oct 2012 (trunk traps), 564 m.a.s.l. (lat. 40.013°long. -4.213°) (MNCN 20.02/17502); 1♂, 20 Aug 2013 (beating), 570 m.a.s.l. (lat. 40.013°long. -4.213°) (MNCN 20.02/17498); 1♀, 27 Sep 2013 (trunk traps), 570 m.a.s.l. (lat. 40.013°long. -4.213°) (MNCN 20.02/17503). All these individuals were deposited in the collection of the National Museum of Natural Sciences (CSIC), Madrid, Spain (MNCN).

#### Additional specimens studied

(Map 1). Spain. Ciudad Real: Piedrabuena, Bullaque river, “Tabla de la Yedra”, 551 m.a.s.l., (lat. 39.041°long. -4.230°), 07 August 97, 1♂ (beating) E. Morano leg (EMH-0899); Isla de Algeciras, P.N. Las Tablas de Daimiel, 617 m.a.s.l, (lat. 39.167°long. -3.661°), 15 July 2015, 2 imm (beating) & 15 October 2015, 1♀ (beating), E. Morano leg (vials n°1522 & 1936). Cáceres: Dehesa Casablanca, Guijo de Granadilla, 405 m.a.s.l. (lat. 40.077°long. -6.097°), 02 November 2016, 1♀, (beating) Morano et al. leg (vial n°1489). Collected by E. Morano in the same year (2013) and locality (Huecas) than the holotype and paratypes but on different months the following specimens have been studied and deposited in the personal collection of Eduardo Morano: February, 1 imm (beating), (lat. 40.013°long. -4.213°); May, 1 imm (beating) (lat. 39.994°long. -4.216°); June, 7 imm (beating) (lat. 39.994°long. -4.216°) & 8 imm (beating) (lat. 40.013°long. -4.213°); July, 17 imm (beating) (lat. 39.994°long. -4.216°) & 7 imm (beating) (lat. 40.013°long. -4.213°); August, 1 imm (beating) (lat. 39.994°long. -4.216°) & 1 imm (beating) (lat. 40.013°long. -4.213°) ; September, 1 ♀ (beating) (lat. 39.994°long. -4.216°) & 1 ♀, 2♂ (trunk traps) (lat. 40.013°long. -4.213°); October, 1 ♂, 1 imm (beating) & 1♀, 1♂ (trunk traps) (lat. 39.994°long. -4.216°).

#### Etymology.

The specific name is dedicated to Dr. Raul Bonal.

#### Diagnosis.

Within the European fauna *Araneusbonali* sp. n. resembles *Gibbaraneagibbosa* (Walckenaer, 1802) due to its colouration (Figure 3) but does not have its characteristic humps on the opisthosoma. The design and greenish coloration of the opisthosoma and the lack of modifications in the male tibias II differentiates the new species from the small sized, and also usually collected in tree canopies, *A.sturmi* (Hahn, 1831) and *A.triguttatus* (Fabricius, 1775). The structure and morphology of the median apophysis of the male palp and the scape and basal plate of the female epigyne of *Araneusbonali* distinguishes it from any similar *Araneus* species.

#### Description.

**Female (holotype).** Measurements of the holotype are shown (ranges for paratypes in parentheses). Total length: 6.0 (5.1–7.2); Prosoma length: 2.4 (2.0–2.5); Prosoma width: 2.5 (1.8–2.5); Opisthosoma length: 4.4 (3.6–4.4); Opisthosoma width: 4.0 (3.2–4.1). Eye diameter: AME: 0.125; ALE: 0.10; PME: 0.10; PLE: 0.075. Distance between eyes: AME – AME: 0.150; AME – ALE: 0.325; PME – PME: 0.125; PME – PLE: 0.375; AME – PME: 0.10; ALE – PLE: 0.05; Height from clypeus to AME: 0.05; Height from clypeus to ALE: 0.05.

Carapace covered by white hairs (Figs 3, 4). Greyish green cephalic area, with a pair of black side bands going from the ocular area to the fovea. Clypeus and sides of the cephalic area dark brown. Glabrous and cream-coloured posterior thoracic region, usually covered by the opisthosoma. Eight eyes in two transverse rows, the four ME arranged in a trapezoid widely separated from two LE. AME distance wider than PME, ME protrude frontally and AME slightly larger than PME (which have a narrow tapetum). Chilum absent. Chelicerae with a proximal boss, their base of the same dark colour than the ocular region and the clypeus. Chelicerae with three teeth in their margins, the median tooth smaller in both cases. Greyish green sternum with dark radial and central bands in the ventral side of the prosoma. Wider than longer labium with a distal white margin. Endites swollen, rebordered, and square, with white internal area, their length only slightly larger than their width. Both have the same colour than the sternum.

Short and relatively stout legs. The first pair the longest and the third the shortest; the second slightly longer than the fourth (Table 1). Dark green coxae and trochanters, rest of segments pale green. Brownish apical third of the femur, base dotted. Patellae usually dark brown, the rest of the segments with dark brown rings at the middle and end. Tarsi without trichobothria.

**Table 1.** Morphological measurements of *Araneusbonali* sp. n. holotype. All measurements are given in millimetres. Leg formula: I>II>IV>III.

Holotype (♀)					
leg	segment	length	leg	segment	length
**I**	**Femur**	3.3	**III**	**Femur**	1.3
	**Patella**	1.2		**Patella**	0.6
	**Tibia**	2,5		**Tibia**	0.7
	**Metatarsus**	2.4		**Metatarsus**	0.7
	**Tarsus**	1.0		**Tarsus**	0.5
	**total**	**10.4**		**total**	**3.8**
**II**	**Femur**	2.1	**IV**	**Femur**	1.7
	**Patella**	0,9		**Patella**	0.7
	**Tibia**	1.4		**Tibia**	1.2
	**Metatarsus**	1.5		**Metatarsus**	1.2
	**Tarsus**	0.6		**Tarsus**	0.6
	**total**	**6.5**		**total**	**5.4**

In females, tibia I and II with 3–4 pairs of lateral spines and 5–6 pairs of ventral spines. Metatarsus I and II with 5–6 spines on the inner side and three basal spines on the outer side. However, the spines are an extremely variable character because they can be lost and may appear in unusual positions or vary with the dimensions of the segments (Grasshoff 1968; Berman and Levi 1971; Carmichael 1973).

Triangular opisthosoma slightly longer than wide, with brown setae. Folium with a black band and a narrow white line that marks the limit between the anterior spots, the posterior humps, and the greenish folium sides. Three pairs of sigillae, the two anterior ones larger. In females, two pairs of anterior humps, much smaller in males. General colouration mimetic with lichens and mosses, difficult to tell the spiders apart when on the oak branches and trunks. White ventral background with two dark lateral spots; two book lungs and an inconspicuous spiracle before the colulus, behind the colulus six spinnerets.

Female genitalia. The scape of the epigyne short and wrinkled (Fig. 2A, B), with setae directed backwards on the surface. Straight scape ending in a spoon-shaped tip and attached to the basal epigynal plate by lateral sclerites. The basal plate or posterior piece as long as the epigyne, with a light tonality and rectangular-shaped, without paired basal lamellae. Ventral genital openings continued internally with the copulatory ducts that connect with the small elliptic spermathecae (Figure 2C).

**Male (Paratypes).** Ranges are shown (and mean values within parentheses) (Table 2). Total length: 3.2–4.3 (3.84); Prosoma length: 1.8–2.3 (2.10); Prosoma width: 1.3–2.1 (1.73); Opisthosoma length: 1.7–2.8 (2.28); Opisthosoma width: 1.2–2.0 (1.76). Eye diameter (average): AME: 0.125; ALE: 0.10; PME: 0.10; PLE: 0.075. Distance between eyes: AME – AME: 0.150; AME – ALE: 0.250; PME – PME: 0.125; PME – PLE: 0.325; AME – PME: 0.10; ALE – PLE: 0.05; Height from clypeus to AME: 0.05; Height from clypeus to ALE: 0.05.

**Table 2.** Morphological measurements of *Araneusbonali* sp. n. paratypes. The values ​​are in millimetres, indicating the minimum, the maximum and, in brackets, the average of each measure. Leg formula: I>II>IV>III.

Dimensions		Paratypes (4 ♂)	Paratypes (3 ♀)	Dimensions		Paratypes (4 ♂)	Paratypes (3 ♀)
**total length**		3.2–4.3 (3.84)	5.1–7.2 (5.74)	**leg**	**segment**	**length**	**length**
**prosoma**	**wide**	1.3–2.1 (1.73)	1.8–2.5 (2.11)	**I**	**Femur**	2.5–3.4 (2.8)	2.5–3.3 (2.8)
	**long**	1.8–2.3 (2.10)	2.0–2.5 (2.23)		**Patella**	0.8–1.4 (1.2)	1.1–1.5 (1.3)
**opisthosoma**	**wide**	1.2–2.0 (1.76)	3.2–4.1 (3.71)		**Tibia**	2.0–2.7 (2.4)	1.8–2.7 (2.3)
	**long**	1.7–2.8 (2.28)	3.6–4.4 (4.10)		**Metatarsus**	2.0–2.5 (2.3)	1.8–2.4 (2.1)
					**Tarsus**	0.8–1.1 (1.0)	0.8–1.0 (0.9)
					**total**	**8.3–10.3 (9.61)**	**8.0–10.7 (9.36)**
				**II**	**Femur**	1.9–2.5 (2.3)	2.0–3.0 (2.4)
					**Patella**	0,7–1.2 (1.0)	0.9–1.3 (1.1)
					**Tibia**	1.2–2.0 (1.7)	1.4–2.2 (1.7)
					**Metatarsus**	1.5–2.0 (1.8)	1.5–1.8 (1.6)
					**Tarsus**	0.7–1.0 (0.8)	0.6–0.9 (0.8)
					**total**	**6.0–8.5 (7.56)**	**6.5–9.0 (7.56)**
				**III**	**Femur**	1.1–1.6 (1,5)	1.3–2.0 (1.6)
					**Patella**	0.4–0.6 (0.5)	0.5–0.8 (0.6)
					**Tibia**	0.7–0.9 (0.8)	0.7–1.0 (0.8)
					**Metatarsus**	0.7–1.0 (0.9)	0.7–1.2 (0.9)
					**Tarsus**	0.5–0.6 (0.6)	0.5–0.7 (0.6)
					**total**	**3.4–4.6 (4.20)**	**3.8–5.7 (4.63)**
				**IV**	**Femur**	1.6–2.5 (2.1)	1.7–2.8 (2.1)
					**Patella**	0.6–0.9 (0.8)	0.6–1.2 (0.9)
					**Tibia**	1.1–1.6 (1.4)	1.2–1.9 (1.5)
					**Metatarsus**	1.0–1.5 (1.4)	1.2–1.9 (1.5)
					**Tarsus**	0.5–0.7 (0.6)	0.6–0.7 (0.6)
					**total**	**5.0–7.2 (6.23)**	**5.4–8.5 (6.67)**

Male general appearance and colouration similar to females (Figure 5) but, according with the large sexual size dimorphism typical of araneids (Hormiga et al. 2000), males are 1.5 times smaller; males slender than females and with a smaller triangular opisthosoma. Light greenish humps, delimited by white lines that continue into the posterior folium. One tooth on the side of the endites. Leg colour identical between sexes, but not their morphology. In males, coxa I has an apical curved hook distally that fits in into the corresponding groove of femur II during copulation. Tibias II curved and armed with four pairs of lateral spines. Metatarsus II with strong spines.

Male genitalia (Figure 1). Palpus with two dorsal macrosetae (on the patella and on the tibia), palpal femur with a small ventral tubercle. Compact genital bulb attached at the base of the cymbium, reduced paracymbium with the standard araneid shape (hook or knob); tegulum covering the base of the palpus. Median apophysis comparatively short on the right side of the radix, with a spur and three distal teeth easy to observe in mesal view (Figure 1B). In this view, the stipe margin is serrated in the area close to the median apophysis. Embolus short, stout and curved towards the conductor and partially covered by the terminal apophysis. Conductor with a swollen distal margin and a large terminal apophysis ending in a fine and blunt tip. The expanded bulb (Figure 1C) shows the presence of radix, stipes, distal haematodocha, and subterminal apophysis.

#### Phylogenetic analyses.

**Figure 6.**
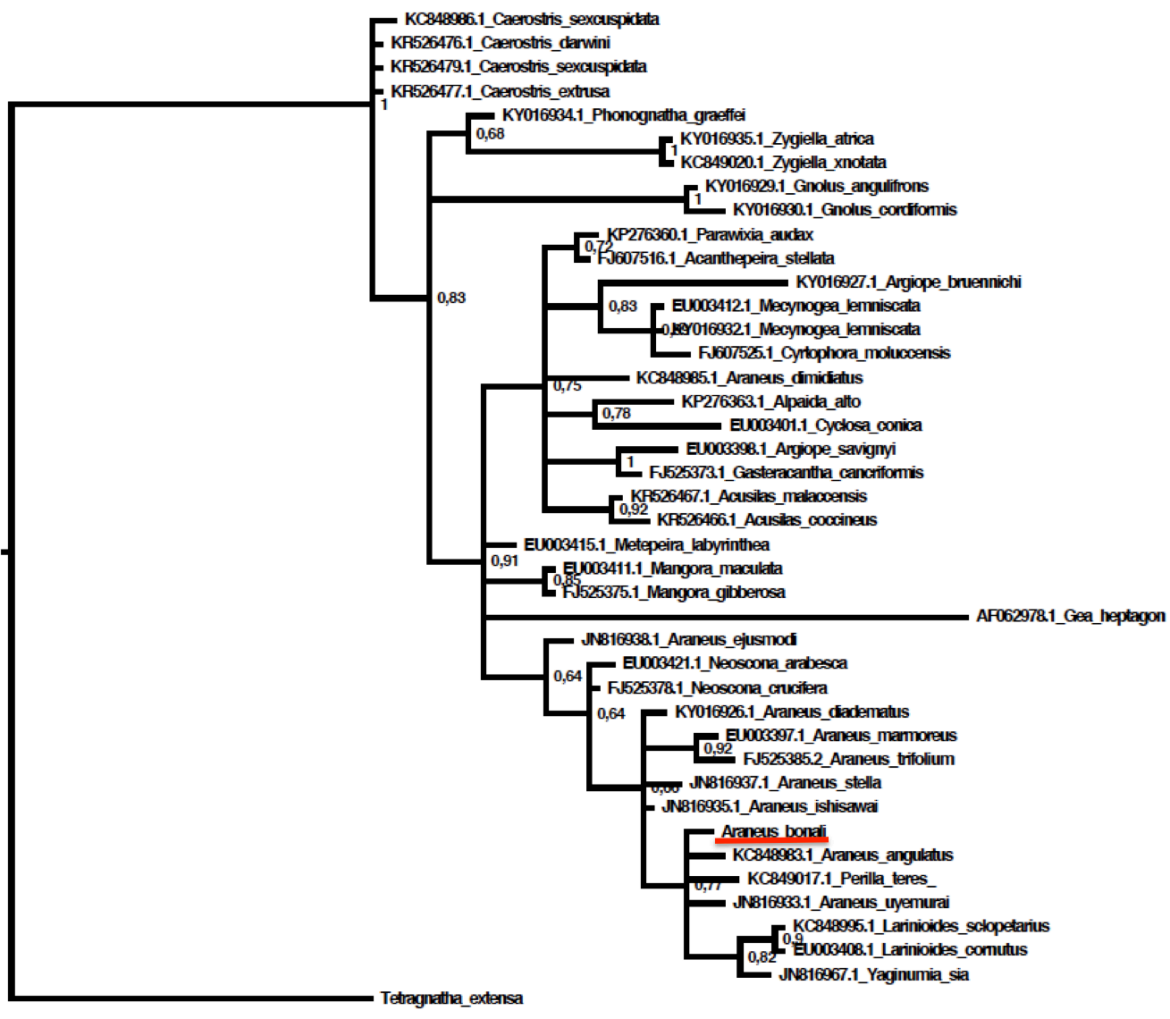
Gene tree of the nuclear gene 28SrRNA. *Araneusbonali* sp. n. underlined in red. Sequences for all species except *Araneusbonali* sp. n. were downloaded from GenBank (accession codes shown besides the species name). Tree topology was inferred using Bayesian inference analyses (GTR + I + Gamma substitution model).

The blast of the nuclear 28SrRNA sequence of the new species recovered *Araneusangulatus* and *Araneusdiadematus* as the most closely related species (sequence similarity 99%). The 28S gene tree (Figure 6) showed that, except *Araneusdimidiatus* (L. Koch, 1871), *Araneus* spp. formed a nonexclusive clade, albeit with low support (PP = 0.64), that also include species from closely related genera such as *Neoscona* Simon, 1864 and *Larinioides*.

**Figure 7.**
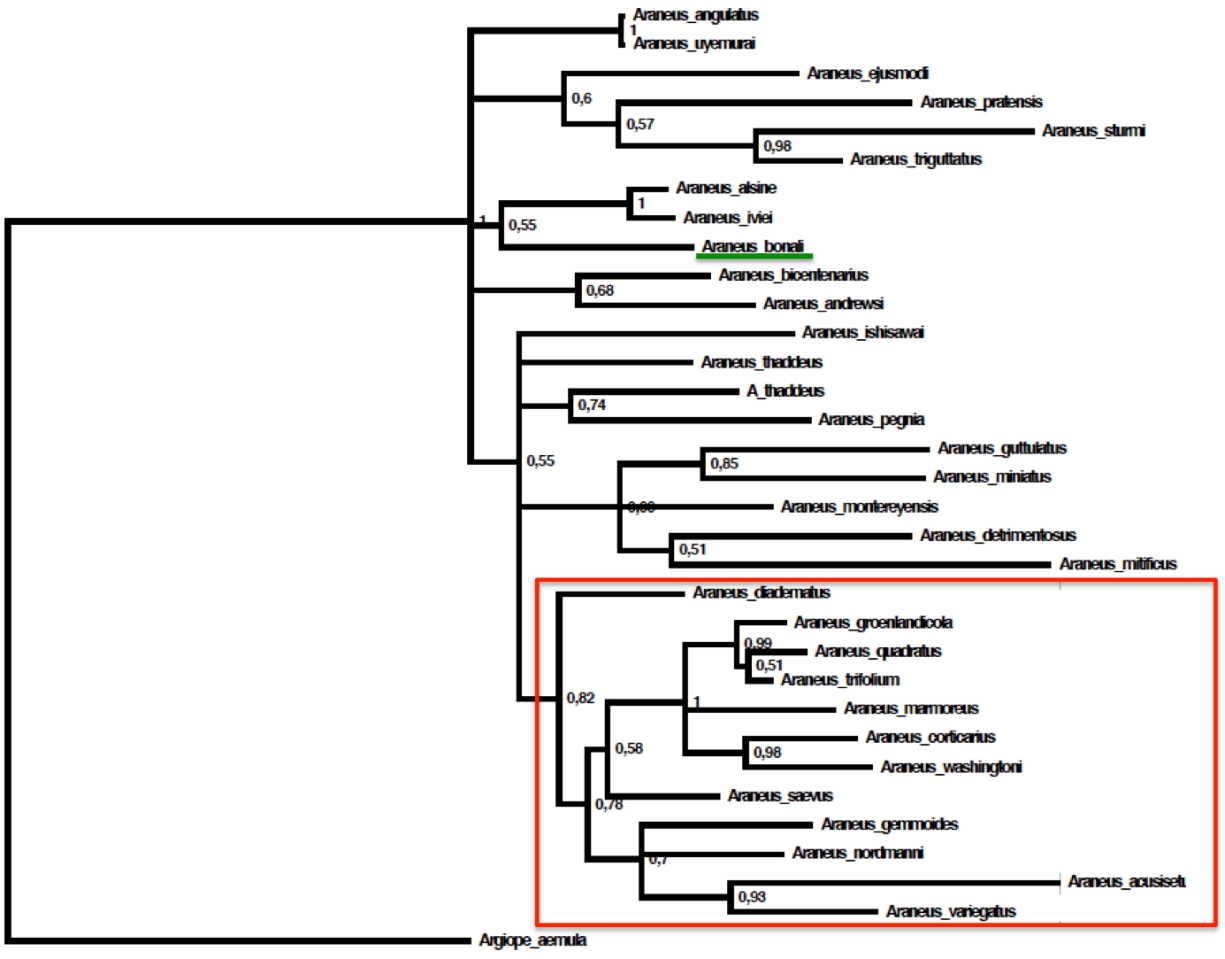
Concatenated mitochondrial cox1 and nuclear 28S genes phylogeny. *Araneusbonali* sp. n. underlined in green within the Holartic *Araneus* sequences available at GenBank or BOLD (accession codes shown in Table 3). The red-coloured frame shows the clade corresponding to the *Araneusdiadematus* group. Tree topology was inferred using Bayesian inference analysis (GTR + I + Gamma substitution model).

The three specimens of the new species analysed yielded the same cox1 haplotype. The lowest uncorrected intraspecific genetic distance was 11.7%, to *Araneusalsine* (Walckenaer, 1802). The average genetic distance among *Araneus* species was 14.9%. The node support values of the concatenated tree were low and most relationships were unresolved. *A.bonali* formed a small clade with *A.iviei* (Archer, 1951) and *A.alsine*, but with a very low support (PP = 0.55) (Figure 7). A clade including *A.diadematus* and eleven additional species, was recovered albeit with low support (PP = 0.82) (Figure 7). The results show that *Araneusangulatus* and the new species are excluded in the *diadematus* group.

**Table 3.** Accession codes for the mitochondrial cytochrome Oxidase I (cox1) and nuclear (28S) gene sequences downloaded from GenBank and BOLD (Barcoding of Life Datasystems) (BOLD sequences in bold characters).

	Cytochrome Oxidase I	28 S
* Araneus acusisetus *	JN817144.1	JN816939.1
* Araneus alsine *	KY268481.1	
* Araneus andrewsi *	**BBUSE10611.COI5P**	
* Araneus angulatus *	JN817138.1	KC848983.1
* Araneus bicentenarius *	**CNSLH311-12.COI-5P**	
* Araneus bonali *	MH517392	MH493065
* Araneus bogotensis *	KR058594.1	
* Araneus corticarius *	KF367835.1	
* Araneus detrimentosus *	**BBUSE122211.COI5P**	
* Araneus diadematus *	KY017584.1	KY016926.1
* Araneus dimidiatus *	KC849065.1	KC848985.1
* Araneus ejusmodi *	JN817143.1	JN816938.1
* Araneus gemmoides *	DQ146861.1	
* Araneus groenlandicola *	GU682824.1	
* Araneus guttulatus *	**CNKOK095-14.COI-5P**	
* Araneus ishisawai *	JN817140.1	JN816935.1
* Araneus iviei *	KM837836.1	
* Araneus marmoreus *	JN817141.1	FJ525384.1
* Araneus miniatus *	**BBUSE179312.COI5P**	
* Araneus mitificus *	KY467247.1	
* Araneus monteryensis *	**BBUSE01411.COI5P**	
* Araneus nordmanii *	GU684587.1	
* Araneus omnicolor *	KP031493.1	
* Araneus pegnia *	**BBUSE144212.COI5P**	
* Araneus poltyoides *	**VAQTB01711.COI5P**	
* Araneus pratensis *	KP653307.1	
* Araneus psittaccinus *	**VAQTB01111.COI5P**	
* Araneus quadratus *	FR775772.1	
* Araneus saevus *	JN309620.1	
* Araneus stella *	JN817142.1	JN816937.1
* Araneus sturmi *	KY269282.1	
* Araneus thaddeus *	HQ924458.1	
* Araneus tijuca *	KT945066.1	
* Araneus trifolium *	GU682571.1	FJ525384.1
* Araneus triguttatus *	KY269635.1	
* Araneus uyemurai *	JN817137.1	JN816933.1
* Araneus variegatus *	JN817139.1	JN816934.1
* Araneus venatrix *	KR058592.1	
* Araneus vincibilis *	KR058596.1	
* Araneus washingtoni *	**ARSO191-08.COI-5P**	
* Araneus workmani *	KR058597.1	
* Argiope aemula *	JX307083.1	DQ018845

#### Habitat distribution and phenology.

**Figure 8.**
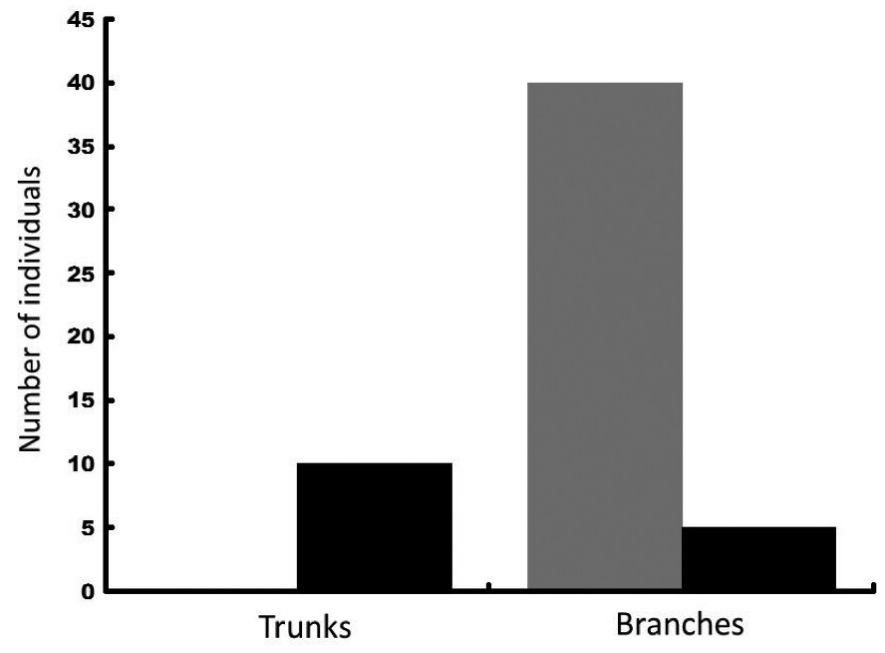
Number of juveniles (grey bars) and adults (black bars) of *Araneusbonali* sp. n. collected on the tree trunks and branches.

**Figure 9.**
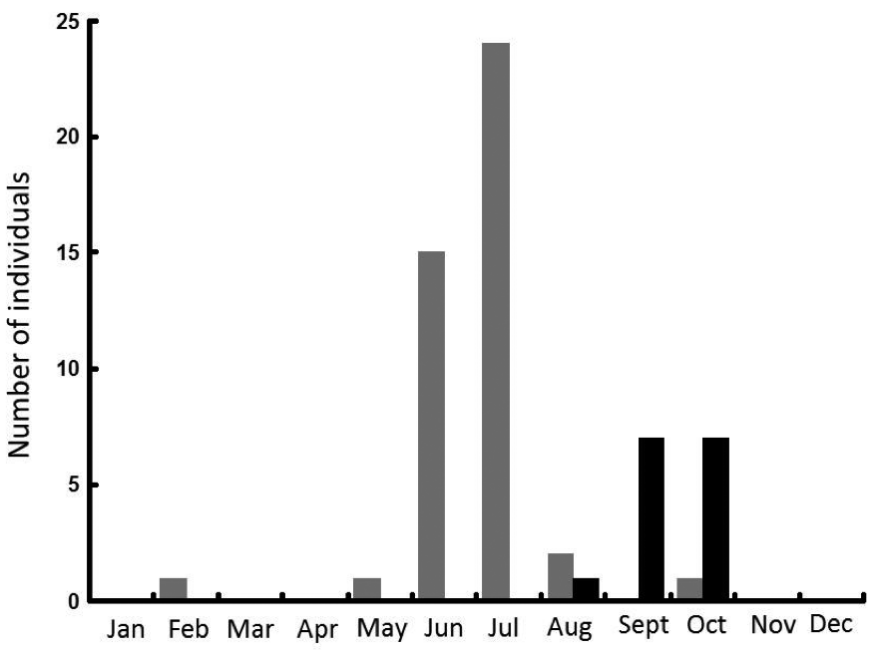
Number of juveniles (grey bars) and adults (black bars) of *Araneusbonali* sp. n. collected throughout the year.

*Araneusbonali* is linked to trees, as not a single individual was collected in ground traps or in grasslands. The distribution patterns differed significantly between juveniles and adults though, as all juveniles (44 individuals) were collected in oak branches whereas most adults (66% of a total of 15 specimens) were captured in trunk traps (Figure 8; Chi = 6.66; df = 1; P < 0.01). Phenology also differed between age classes (Chi = 135; df = 3; P < 0.001). With the exception of one capture in February, the bulk of juveniles appeared in May and matured throughout spring and summer. Adult presence and reproduction was concentrated in the months of September and October and none were caught in winter (Figure 9).

*Araneusbonali* was collected in 18 of the 24 holm oaks sampled. The number of juveniles and adults caught at each oak were significantly related (F_1, 22_ = 31.41; P < 0.001). The total number of individuals trapped at each tree was unrelated with canopy surface (GLM Estimate = 0.001; Z = 0.35; P = 0.72) or the pairwise spatial distance between holm oaks (Mantel test R = -0.04; P = 0.62).

## Discussion

*Araneusbonali* sp. n. exhibits a combination of somatic and genital characters typical of *Araneus* according to the cladistic analyses performed by Scharff and Coddington (1997), which established the intergeneric relationships within the family Araneidae. These traits include a hairy carapace in both sexes and in males the presence of an endite tooth, coxa I with hook and curved tibia II with strong macrosetae. Male genitalia were thoroughly analysed (including the expanded bulb) and has two patellar setae, a median apophysis with spines and hooks, stipes and two apophysis (subterminal and terminal) separated from distal hematodocha. The cap on the tip of the embolus present in virgin males was not found in the individuals analysed. Females showed the pockets near the tip of the epigynal scape characteristic of the genus (Levi 1991; Scharff and Coddington 1997). The molecular analyses supported genus membership of the new species by identifying *Araneusangulatus* (the type species of the genus) and the common *Araneusdiadematus* as the two closest species, and the 28S gene tree showed the new species within the *Araneus* group along with other species of the genus.

The morphology of the male and female genitalia clearly distinguished the new species from any other *Araneus* species. Moreover, large uncorrected genetic distance to the closest relative (11.7%), further confirms its species status (Ratnasingham and Herbert 2007). *Araneusbonali* colouration is similar to that of *Araneuscirce* (Audouin, 1826), however, the presence of the latter species in the Iberian Peninsula has not been confirmed (see discussion about the taxonomic status of Iberian *Araneus* spp. below). Moreover, *A.circe* is a large orbweaver (female length more than 20 mm), about three times bigger than *A.bonali*. The new species resembles another three small European araneids, namely *Gibbaraneagibbosa*, *Araneussturmi* (Hahn, 1831) and *Araneustriguttatus* (Fabricius, 1775) and, like them, inhabits trees. It could be confused with the first species due to the greenish body (Figure 1). However, the new species lacks the prominent humps on the opisthosoma typical of *G.gibbosa*. Their genitalia are also different, in *G.gibbosa* male median apophysis has a broad base and a tapering, curved spur and the scape of female epigyne finishes in a characteristic spoon-shaped tip. Regarding the other two species, the lichen-like and greenish grey opisthosoma of *Araneusbonali* distinguishes it from the orangish red colour of *A.sturmi* and *A.triguttatus*. In addition, the structure and morphology of the copulatory organs (especially the straight scape of female epigyne and the three small teeth of the median apophysis of male palps in *A.bonali*) allow their differentiation.

**Figure 10.**
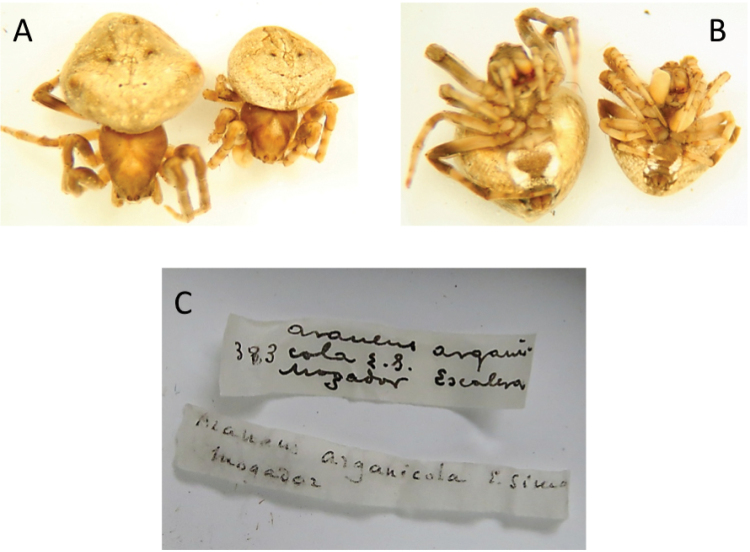
*Araneusarganicola* syntype specimens. **A** dorsal view **B** ventral view **C** data labels.

A thorough revision of the literature was carried out including reports and descriptions of *Araneus* in Northern Africa (Audouin 1826; Cambridge 1872; Thorell 1875; Cambrigde, 1876; Pavesi 1880; Simon 1885; Lucas 1846; Simon 1899; Simon 1908; Simon 1909; Caporiacco 1934; Denis, 1945; Jocqué 1997) and three species not cited in Europe were found, namely *Araneusarganicola* (Simon, 1909), *Araneusklaptoczi* (Simon, 1908) and *Araneusv-notatus* (Thorell, 1875). The specimens of *A.klapotczi* and *A.v-notatus* could not be examined but, based on the original descriptions, they cannot be mistaken with *A.bonali*. In the first case, the description matches that of the modern genera *Pararaneus* Caporiacco, 1940, *Larinia* Simon, 1874 or *Siwa* Grasshoff, 1970. In the case *A.v-notatus*, the scape of the female genitalia has the characteristic “S” shape of *Araneussturmi*. In spite of the different body colour, Simon (1929) included this species as a variety of *A.sturmi* when it was collected in France. The syntypes of *A.arganicola* deposited at the collection of the National Museum of Natural Sciences (Madrid) (voucher MNCN 20.02/12093) could be examined. They were collected by Martínez Escalera in Mogador (Morocco) in the nineteenth century. After a detailed inspection it was concluded that they were two subadults (♀ and ♂), as can be appreciated in Figure 10 where the outlines of female epigyne and swollen male palps can be observed. Both specimens probably belong to the species *Neosconasubfusca* (CL Koch, 1837). Besides these specimens, there were other two collected by the same person in Morocco (vouchers and collecting sites: MNCN 20.02/12120 -Mogador- and MNCN 20.12/12115 -Tanger-). They were labelled as *A.circe* but, after a detailed morphological analysis, we concluded that they were two females de *A.angulatus*.

The 28S gene tree confirmed that the only non-Holartic species that was in the analysis (28S sequence available), namely the Australian *Araneusdimidiatus*, does not belong in the *Araneus* spp. group (fig. 28S tree) (Framenau 2012) and supports our decision of not including them in further phylogenetic analyses. The *Araneus* clade included species of other genera such as *Larinioides* and *Neoscona*, probably due to the lack of informative characters in this short fragment. The concatenated phylogenetic tree of the Holartic *Araneus* spp. recovered a low supported clade (PP = 0.82) that grouped *Araneusdiadematus* with another eleven species. Neither *Araneusbonali* nor *Araneusangulatus* or *Araneussturmi* seem to belong to this group. Nonetheless, the poor resolution of our tree caution against drawing any major conclusions. New phylogenetic analyses based on a more thorough taxonomic sampling and a larger combination of molecular markers would be required to confirm some of our results and provide a fully resolved phylogenetic hypothesis for the genus and its close allies.

The new species is a tree-specialist: in addition to the more intensively studied area, it was found in holm oaks in the other localities of central and western Spain sampled. However, microhabitat selection did differ among life stages. While juveniles were in all cases collected in the branches, adults were more often caught in trunk traps. The differences in colouration might explain such a contrast in some extent. Juveniles are greenish, similar to oak new shoots, whereas adults show a greyish green colour remarkably similar to the lichens that cover the oak trunks (Figure 3). This mimicry might help them to remain undetected by prey and/or avoid predators, as has been found in other species (Théry and Casas 2002). This lichen-like appearance is not unique to the new species but also shared by another closely related species, namely the American *Araneusbicentenarius* (McCook, 1888) (Figure 6), so called `giant lichen orbweaver´, which was collected on trees among lichens (Levi 1971). The new small Iberian species would thus have a “giant” counterpart on the other side of the Atlantic, as the females of *A.bicentenarius* (total length 28 mm) are more than four times larger than those of *A.bonali* (total length 6 mm).

The number of individuals of the new species collected at each tree was not spatially correlated, what suggests that *A.bonali* is able to disperse even in a landscape in which trees are isolated. Its small size may favour ballooning dispersal, a behaviour previously described in different araneids (Bell et al. 2005). Nonetheless, adult and juvenile numbers were significantly related at the tree level, what also points to the relative importance of local reproduction. With the exception of one individual in February, juveniles were present from mid-spring onwards and the first adults did not appear until late summer. Based on Schaeffer (1977) and Ysnel and Canard (1986) classification, *A.bonali* would be an autumn stenocorus species, characterized by a short biological cycle with a brief adult presence between late summer and early autumn and a wintering period as egg. This life-cycle phenology is the most common in European *Araneus* spp. (Nentwig et al. 2017), although in the new species adult presence extends a little more into early autumn, probably due to the warmer temperatures in the Mediterranean climate.

### The status of *Araneus* spp. in the Iberian Peninsula: updating the species list

The bibliographical review and the revision of the material from several collections allowed evaluation of the taxonomic status and update the list of the Iberian *Araneus* spp. So far, there were several records of the following seven species in the Iberian Peninsula: *A.angulatus, A.diadematus*, *A.marmoreus* Clerck, 1757, *A.pallidus* (Olivier, 1789), *A.quadratus* Clerck, 1757, *A.sturmi*, and *A.triguttatus* (Morano, Carrillo & Cardoso, 2014). Another three species, namely *Araneuscirce*, *A.grossus* (C. L. Koch, 1844) and *Epeiraspinivulva* (Dufour, 1835), have been cited in the past, but either the records need to be confirmed or their taxonomic status carefully reviewed.

The presence of *Araneuscirce* (Pozuelo de Calatrava & Fuente, 1898) and *A.grossus* (Coimbra & Bacelar, 1928), species well known in other European regions, needs confirmation as only two records from old bibliographical references are available for the Iberian Peninsula. The taxonomic status of *Epeiraspinivulva* is doubtful, and very probably corresponds to a synonymy. Léon Dufour describes a female of *Epeiraspinivulva* in Sagunto (Valencia) (Dufour 1835), a species also cited in the Portuguese locality of Povoa de Varzim (Bacelar 1927). The type material of this species was insufficiently described and, unfortunately, it could not be found after contacting the museum where it was deposited. Simon (1874) considers it valid, including among the synonyms *Epeiravulpina* (Hahn, 1835). In 1929, Simon mentions it in the third footnote (p 756): “est absolument impossible de savoir ce que peut être l’*Epeiraspinivulva* of Léon Dufour”, including it as a synonym of the species *Araneusangulatus*. Bonnet (1955) separates them as valid species and on footnote (p. 631) indicates that “Cette espece a été homologuée avec doute, avec l’*Epeiraspinivulva* de Dufour”. Nonetheless, the current World Spider Catalog considers *Epeiraspinivulva* a synonym of *A.vulpinus* (Hahn, 1834). After these doubts, it should be considered as “*nomen nudum*”, because Dufour only vaguely describes the scape of the epigyne and the colour of the specimen. With so little information it is not possible to differentiate it from species like *A.angulatus*, *A.circe*, *A.grossus* or even *A.diadematus*.

Finally, in his articles from the beginning of the 20^th^ century, Franganillo described a series of varieties of different species, and even new species of *Araneus*, using ambiguous descriptions lacking illustrations (Franganillo 1909, 1910, 1913, 1918a, b). Recent reviews of bibliographical references and collection materials have concluded that those species are really synonymies of known species (Breitling et al. 2016). It was also Franganillo (1910) who, following Emerton (1884), determined a specimen collected in Spain as “*E.thadeus* Hent” in his article entitled “Arañas de la desembocadura del Miño” (Franganillo 1909). He briefly describes the epigyne of the specimen as “a very short and wide hook, quite convex, like a lid over the genital openings; rounded in its distal extreme and with a small flap in the middle”, the colouration of the individual is described as “greenish”. Neither the morphology of the epygine nor the body colour match those of *Araneusthaddeus* Hentz (Levi 1973, Dondale et al. 2003). Unfortunately, the specimen is probably lost, as it could not be found in the most recent revision of Franganillo´s collection (Breitling et al. 2016). In our view, Franganillo´s description could correspond to a specimen of *Araniellacucurbitina* (Clerck, 1757) or *Araniellaopistographa* (Kulczyński, 1905), both present in the Iberian Peninsula.

## Conclusions

The morphological and genetic analyses confirm that *Araneusbonali* is a new species, hence, the list of *Araneus* in the Iberian Peninsula now numbers eight species: *A.angulatus*, *A.bonali*, *A.diadematus*, *A.marmoreus*, *A.pallidus*, *A.quadratus*, *A.sturmi*, and *A.triguttatus*. The inclusion of *A.circe* and *A.grossus* remains to be confirmed.

The geographical distribution of the new species remains to be fully delimited to confirm whether it is widespread or, similar to other species (e.g., *Araneuspallidus*), its distribution is restricted to the western Mediterranean Basin. The fact that it has not been collected elsewhere in Europe before suggests a potential small geographical range, however, it is true that due to its habitat preferences it may have gone unnoticed, as tree trunks are not so frequently sampled. In the case of Spain, this is the second new species collected in isolated holm oaks within croplands (the first, *Cheiracanthiumilicis* was sampled in the same study site; see Morano and Bonal 2016). Hence, sampling efforts on tree branches and trunks in further spider field surveys are encouraged. From a conservation perspective, the preservation of isolated trees and forest patches in croplands should be high in the agenda of nature management policies (Guevara et al. 2005, Manning et al. 2006, Bonal et al. 2012).

## Supplementary Material

XML Treatment for Araneus bonali
